# Enhancing cannabinoid bioavailability: a crossover study comparing a novel self-nanoemulsifying drug delivery system and a commercial oil-based formulation

**DOI:** 10.1186/s42238-025-00294-8

**Published:** 2025-06-13

**Authors:** Vered Hermush, Nisim Mizrahi, Tal Brodezky, Rafael Ezra

**Affiliations:** 1 Geriatric Wing, Laniado Hospital, Netanya, Israel; 2 Ariel School of Medicine, Ariel, Israel; 3 Pharmaceutical Association of Israel, Tel Aviv, Israel

**Keywords:** Cannabinoid bioavailability, Self-Nanoemulsifying drug delivery systems, SNEDDS, Pharmacokinetics, Delta-9-tetrahydrocannabinol, THC, Cannabidiol, CBD, 11-OH-THC, 7-OH-CBD

## Abstract

**Purpose:**

The oral bioavailability of cannabinoids is limited due to extensive first-pass metabolism, reducing their therapeutic efficacy. This study aimed to evaluate the pharmacokinetics and relative bioavailability of cannabinoids delivered via delta-9-tetrahydrocannabinol/cannabidiol self-emulsifying (THC/CBD-SE) powder, a self-nanoemulsifying drug delivery system, compared to standard oil-based drops.

**Methods:**

Fourteen healthy volunteers (3 men, 11 women) participated in a crossover study. Each received a single oral doses of 8 mg THC and 8 mg CBD in both formulations, with a 30-day washout period between treatments. Blood samples were collected at specified intervals post-administration to assess pharmacokinetic parameters, including maximum plasma concentration (Cmax) and time to reach Cmax (Tmax).

**Results:**

THC/CBD-SE Powder significantly enhanced Cmax for THC (32.79 ± 44.37 ng/mL) and its metabolite 11-OH-THC (10.91 ± 6.64 ng/mL) compared to oil-based drops (THC: 10.17 ± 11.41 ng/mL; 11-OH-THC: 4.64 ± 2.55 ng/mL). Similarly, Cmax for 7-OH-CBD was higher with THC/CBD-SE Powder (2.38 ± 1.63 ng/mL vs. 0.86 ± 0.56 ng/mL). Tmax for 11-OH-THC and 7-OH-CBD was shorter with THC/CBD-SE Powder (0.86 ± 0.36 h vs. 4.54 ± 3.44 h and 1.11 ± 0.59 h vs. 4.68 ± 3.38 h, respectively), indicating a faster onset of action. The THC/CBD-SE Powder exhibited over double the relative bioavailability of cannabinoids compared to oil drops, suggesting improved absorption and rapid onset. Both formulations were well tolerated with no serious adverse events.

**Conclusion:**

THC/CBD-SE Powder significantly improves cannabinoid bioavailability and absorption rates compared to oil-based drops, offering a promising oral delivery method for enhanced therapeutic potential.

## Background

*Cannabis sativa*, a plant with a rich history of medicinal use and scientific exploration, boasts a complex phytochemical composition (Andre et al. 2016). Among its constituents, delta-9-tetrahydrocannabinol (THC) and cannabidiol (CBD) have garnered significant attention due to their pharmacological properties (Bilbao and Spanagel 2022; Costa 2007; Mandolini et al. 2018). THC, the primary psychoactive component, along with its active metabolite, 11-hydroxy-delta-9-tetrahydrocannabinol (11-OH-THC), is renowned for its euphoric effects and analgesic properties (Ashton 2001; Ross 2023). Meanwhile, CBD and its major active metabolite, 7-hydroxy-cannabidiol (7-OH-CBD), have been investigated for their anxiolytic, anticonvulsant, and anti-inflammatory potential, offering therapeutic prospects for diverse conditions including epilepsy, anxiety disorders, pain syndromes, inflammatory diseases, and schizophrenia (Omotayo et al. 2024; Gurgenci et al. 2023; Sholler et al. 2020).

However, the pharmacokinetics of cannabinoids present considerable challenges in clinical settings. The lipophilic nature of these compounds results in poor aqueous solubility and suboptimal absorption within the gastrointestinal tract, leading to low oral bioavailability. For instance, the bioavailability of CBD is approximately 6% under fasting conditions, enhanced by lipid-rich meals (Perucca and Bialer 2020). Furthermore, first-pass hepatic metabolism significantly impacts systemic availability, with cytochrome P450 enzymes metabolizing a substantial proportion of ingested cannabinoids (Perucca and Bialer 2020; Bansal et al. 2022). Approximately 70–75% of an orally administered dose of CBD undergoes hepatic metabolism before reaching the systemic circulation (Perucca and Bialer 2020). THC, on the other hand, exhibits variable bioavailability depending on the route of administration, with oral intake achieving only 4–20% bioavailability due to extensive hepatic metabolism (O’Brien and Blair 2021). Pulmonary routes, such as smoking or vaporizing, bypass first-pass metabolism, allowing higher bioavailability but also introducing respiratory risks (O’Brien and Blair 2021; Page et al. 2020). Inhalation methods provide rapid onset but can cause respiratory complications, including chronic bronchitis and increased susceptibility to infections (Tan and Sin 2018; Kaplan 2021; Tetrault et al. 2007). Furthermore, the combustion process releases carcinogens and other harmful compounds. Although vaporization reduces exposure to combustion products, it has been associated with acute lung injury and may cause tachycardia, impaired coordination, and cognitive side effects (Kaplan 2021; Spindle et al. 2018).

Various strategies have been explored to enhance cannabinoid bioavailability, with lipid-based formulations, emulsions, and encapsulations showing promise for improving absorption and overall systemic delivery (O’Sullivan et al. 2024; Varadi et al. 2023). Among these, self-nanoemulsifying drug delivery systems (SNEDDS) have garnered significant interest as potential oral delivery methods for lipophilic drugs, such as cannabinoids (Buya et al. 2020). SNEDDS formulations, which consist of isotropic mixtures of oils, surfactants, and co-surfactants, create oil-in-water nanoemulsions upon contact with gastrointestinal fluids, thereby enhancing drug absorption through increased surface area (Ahmad et al. 2020). Recent studies indicate that SNEDDS can enhance intestinal membrane permeability and protect against gastrointestinal degradation, making them suitable for various bioactive compounds (Ding et al. 2016; Hetenyi et al. 2017; Lin et al. 2023; Rathore et al. 2023).

While prior studies have examined SNEDDS-based cannabinoid formulations in capsule form (Atsmon et al. 2018; Izgelov et al. 2020; Knaub et al. 2019; Cherniakov et al. 2017a, 2017b), this study investigates a THC/CBD self-emulsified powder (THC/CBD-SE Powder), a SNEDDS-based formulation developed by Capsoil Technologies Ltd. This powder is created through a multi-step process involving pre-emulsification, high-pressure nano-emulsification, and freeze-drying with a sugar matrix. The resulting water-soluble powder encapsulates nano-micelles, preserving the cannabinoid profile without chemical alteration. Unlike traditional SNEDDS, which rely on partial emulsification in aqueous environments, THC/CBD-SE Powder achieves full emulsification, enabling high cannabinoid loading (30–60% w/w) while addressing common SNEDDS limitations, such as high surfactant requirements, organic solvents, and low active pharmaceutical ingredient concentrations (Buya et al. 2020; Salawi 2022). The powder format may improve solubility, stability, and dosing precision, offering flexibility for pharmaceutical applications, with added benefits of extended shelf life and consistent bioavailability.

Our study provides a direct comparison of THC/CBD-SE Powder with a commercially available oil-based formulation, analyzing the pharmacokinetics of both THC and CBD, along with their metabolites 11-OH-THC and 7-OH-CBD. We aim to evaluate the pharmacokinetic properties and relative bioavailability of cannabinoids delivered via THC/CBD-SE Powder versus standard oil drops. We hypothesize that the nanoemulsion properties of THC/CBD-SE Powder will improve cannabinoid solubility and absorption, resulting in faster and more effective bioavailability.

## Methods

### Study subjects

From August to November 2023, a total of 14 subjects were pre-screened for eligibility. According to the inclusion criteria, subjects were aged 21–65 years and confirmed to be in good health based on medical history, physical examination, electrocardiogram (ECG), vital signs, and biochemistry and hematology tests. Female subjects underwent pregnancy testing. Participants were excluded if they had allergies to THC/CBD-like compounds, medical conditions affecting the liver, kidneys, heart, blood vessels, or immune system, or if they used chronic medications or any medications within 24 h prior to treatment. Other exclusions included psychiatric or substance abuse disorders, a history of depression, anxiety, high or low blood pressure, diabetes, physical limitations, recent participation in another investigational study, cannabis use or a positive THC test, and pregnancy or lactation. All participants provided written consent and applied for a cannabis treatment license. All 14 subjects (3 men, 11 women) completed the study without significant protocol deviations.

### Study design

The study employed a fixed-sequence crossover design with two treatment periods separated by a 30-day washout period. All fourteen healthy volunteers first received a single dose of cannabis, standardized to 8 mg THC and 8 mg CBD, as THC/CBD-SE Powder, followed by a second dose of the same cannabinoids in commercial oil-based drops. In preparation for each intervention, subjects fasted for 8 h. Additionally, before the administration of the study drug, subjects were provided with a standardized snack consisting of two cookies and 200 mL of apple juice, totalling approximately 180 kcal and 4 g of fat. The snack was intended to minimize food-drug interactions and prevent hypoglycemia.

Each subject attended two visits. During both Visit 1 (up to 28 days after screening) and Visit 2 (30 days ± 10 days after Visit 1), participants provided a baseline urine sample for THC presence verification, followed by various assessments including physical examination, vital signs, complete blood count, and biochemistry. After consuming the snack, subjects received a single dose of either THC/CBD-SE Powder (Visit 1) or commercial oil-based drops (Visit 2). Blood samples were collected at baseline and at 0.5, 1, 1.5, 2, 4, 6, 8, and 10 h post-administration for pharmacokinetic analysis.

The primary objective was to evaluate the pharmacokinetic profile and relative bioavailability of cannabinoids in the THC/CBD-SE Powder compared to the commercial oil-based formulation. Pharmacokinetic parameters included peak concentration (Cmax), area under the curve (AUC), and time to peak concentration (Tmax), and are presented as mean ± standard deviation (SD). The secondary objective was to assess the tolerability and safety of the THC/CBD-SE Powder versus the commercial oil through adverse event monitoring. Additionally, subjects rated their euphoria sensation using a numeric scale from 0 to 10, where 0 indicated no effect, 5 indicated a moderate effect, and 10 indicated a very intense feeling. The score was documented at different time points post-administration.

### Intervention

Subjects received a single oral dose of either THC/CBD-SE Powder or a commercial oil-based formulation under medical supervision. The THC/CBD-SE Powder formulation consisted of 500 mg of a mixture containing 43% sucrose, 29% maltodextrin, 16% cannabis oil (Medical Grade T10/C10 oil, 8 mg THC and 8 mg CBD), 9% olive oil, and 3% glycyrrhizic acid (ammonium salt) as a natural emulsifier. The cannabis oil was extracted from inflorescences grown by Tikun Olam Ltd., in accordance with Good Manufacturing Practice (GMP) and certified quality systems (ISO 9001 and HACCP). The oil was transformed into powder using a proprietary physical process that included pre-emulsification, high-pressure nano-emulsification, and freeze-drying with a matrix of disaccharides and polysaccharides. This resulted in a water-soluble powder encapsulating nano-micelles, which release upon reconstitution in water without requiring additional energy. Particle size analysis using a Malvern-Zetasizer showed an initial average droplet size of 158 nm with a polydispersity index (PDI) of 0.21. Approximately 80% of particles were within the 80–250 nm range, consistent with typical nanoemulsion characteristics. After freeze-drying and re-dispersion, the particle size remained stable at 157 nm with a PDI of 0.19.

To administer the THC/CBD-SE Powder, the contents of a sachet were dissolved in 125 ml of filtered, room-temperature water and stirred until a slightly hazy solution formed. Subjects then consumed the solution, followed by an additional 125 ml of water to rinse the glass and ensure the full dose was taken. The commercial oil-based formulation (containing the same cannabis oil - Medical Grade T10/C10 oil) was administered sublingually. Subjects placed two drops (8 mg THC and 8 mg CBD) under the tongue, held it for 1–2 min, and avoided consuming fluids for 15 min to ensure absorption.

### Sample analysis

Blood samples were collected in EDTA Vacutainer tubes with Plasma Sep Clot Activator. Samples were centrifuged at 4 °C at 1000 x g for 20 min within one hour of collection. Plasma fractions were aliquoted into 1.5-mL Eppendorf tubes, stored frozen at -80 °C, and analyzed at Cannasoul Analytics Lab, Israel. Plasma samples were transported at up to -20 °C in a monitored package. The exact sampling time relative to study products administration was recorded in the Case Report Form. The bioanalytical assay used for quantification of cannabinoids and their metabolites was developed and validated by Cannasoul Analytics under Good Clinical Laboratory Practice standards.

### Analysis software and statistical analysis

All patients were analyzed. All continuous variables were displayed as mean and SD. Analyses stratified by cannabinoids and metabolites compared drug delivery systems using paired Wilcoxon or t-tests as applicable. Relative bioavailability was analyzed using AUC, which was calculated from time zero to 10 h post-dose (AUC₀–₁₀). All plasma concentration values were above the lower limit of quantitation (LLOQ), and no imputation or censoring was required. All available time points were used in AUC calculation and in generating PK profile plots. All statistical analyses were two-tailed tests and significance was set at 5%. Results were analyzed using SPSS Version 23.0 (IBM, SPSS Inc., Chicago, Ill., USA).

## Results

### Study cohort

The study was conducted in 2023 at Laniado Hospital in Israel. Fourteen healthy volunteers (3 men, 11 women; 78.6% female) completed the study, with a mean age of 43.57 years (range: 25–58) and a mean BMI of 27.2 kg/m². Vital signs, physical examinations, and ECG were within normal ranges. Participants were invited for Visit 1 over two consecutive days: five on the first day and nine on the second. A month later, all subjects returned for crossover to the second drug delivery system (Visit 2). There was no differentiation between patients’ recruitment days.

### Pharmacokinetics

The study compared the pharmacokinetic characteristics of two cannabis delivery systems: THC/CBD-SE Powder and oil-based drops. The drug delivery system did not alter the overall kinetic profiles of the cannabinoids, which exhibited two concentration peaks, while their metabolites showed a single release profile. All tested cannabinoids (THC and CBD) and their active metabolites (11-OH-THC and 7-OH-CBD) exhibited more than double the plasma concentrations (Cmax) with THC/CBD-SE Powder compared to oil drops and reached maximal concentrations faster (shorter Tmax). The relative bioavailability was significantly higher with THC/CBD-SE Powder.

#### THC and 11-OH-THC

The THC/CBD-SE Powder formulation resulted in significantly higher maximum plasma concentrations for both THC (32.79 ± 44.37 ng/mL vs. 10.17 ± 11.41 ng/mL, *p* = 0.044) and its active metabolite 11-OH-THC (10.91 ± 6.64 ng/mL vs. 4.64 ± 2.55 ng/mL, *p* = 0.002). The Tmax for 11-OH-THC was significantly shorter with the THC/CBD-SE Powder (0.86 ± 0.36 h vs. 4.54 ± 3.44 h, *p* = 0.002), indicating a more rapid absorption. For THC, the Tmax was shorter with THC/CBD-SE Powder (2.89 ± 3.56 h) compared to the oil drops (4.25 ± 3.11 h), although this difference was not statistically significant (*p* = 0.393).

Moreover, THC via THC/CBD-SE Powder reached the Cmax achieved by the oil drops formulation in just 54 min, compared to 4.3 h for the oil drops to reach their own Cmax. Similarly, 11-OH-THC via THC/CBD-SE Powder reached the oil drops’ Cmax in 22 min, compared to 4.5 h for the oil drops, demonstrating the rapid attainment of peak concentrations despite the higher Cmax with THC/CBD-SE Powder.

**Fig. 1:**
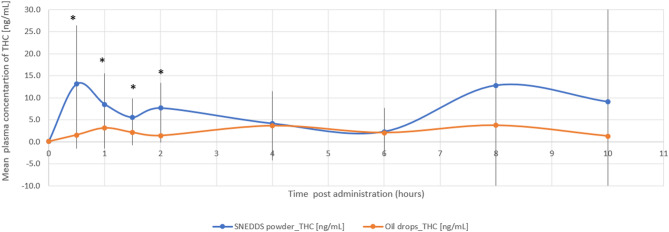
Mean plasma concentration of THC over time following cannabis administration via THC/CBD-SE Powder (blue) and oil drops (orange) (*N* = 14). Vertical bars represent the standard deviation. *p < 0.05

**Fig. 2:**
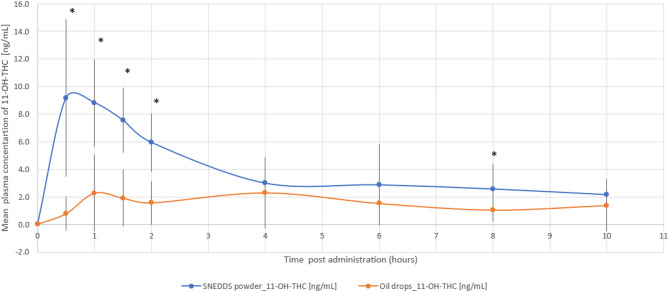
Mean plasma concentration of 11-OH-THC over time following cannabis administration via THC/CBD-SE Powder (blue) and oil drops (orange) (*N* = 14). Vertical bars represent the standard deviation. *p < 0.05

The relative bioavailability of THC and 11-OH-THC was 2.9-fold (*p* = 0.022) and 2.5-fold (*p* = 0.006) higher with the THC/CBD-SE Powder, respectively. Table 1 provides a detailed summary of the pharmacokinetic parameters for THC and 11-OH-THC, underscoring the superior absorption and bioavailability of the THC/CBD-SE Powder compared to the oil drops. Figures 1 and 2 illustrate the mean plasma concentrations of THC and 11-OH-THC per hour post-administration for both formulations, further highlighting the enhanced pharmacokinetic profiles of the THC/CBD-SE Powder formulation.

**Table 1:** Pharmacokinetic parameters of THC and 11-OH-THC

Variable	THC/CBD-SE Powder Group (*N* = 14) Mean ± SD or %	Oil Drops Group (*N* = 14) Mean ± SD or %	*p*-value
THC			
Cmax (ng/mL)	32.79 ± 44.37	10.17 ± 11.41	**0.044**
Tmax (h)	2.89 ± 3.56	4.25 ± 3.11	0.393
AUC (ng·h/mL)	70.59 ± 101.20	24.63 ± 30.88	**0.022**
Relative Bioavailability	100%	35%	-
11-OH-THC			
Cmax (ng/mL)	10.91 ± 6.64	4.65 ± 2.55	**0.002**
Tmax (h)	0.86 ± 0.36	4.54 ± 3.44	**0.002**
AUC (ng·h/mL)	38.31 ± 22.30	15.32 ± 8.25	**0.006**
Relative Bioavailability	100%	40%	**-**

#### CBD and 7-OH-CBD

The THC/CBD-SE Powder formulation resulted in higher maximum plasma concentrations for CBD (7.23 ± 8.54 ng/mL vs. 3.30 ± 3.49 ng/mL, *p* = 0.121) and its active metabolite 7-OH-CBD (2.38 ± 1.63 ng/mL vs. 0.86 ± 0.56 ng/mL, *p* = 0.002). The Tmax for 7-OH-CBD was significantly shorter with the THC/CBD-SE Powder (1.11 ± 0.59 h vs. 4.68 ± 3.38 h, *p* = 0.002), indicating more rapid absorption. For CBD, the Tmax was shorter with THC/CBD-SE Powder (2.57 ± 3.17 h) compared to the oil drops (4.57 ± 3.42 h), although this difference was not statistically significant (*p* = 0.175).

Moreover, CBD via THC/CBD-SE Powder reached the Cmax achieved by the oil drops formulation in just 70 min compared to 4.57 h for the oil drops to reach their own Cmax. Similarly, 7-OH-CBD via THC/CBD-SE Powder reached the oil drops’ Cmax in 24 min compared to 4.68 h for the oil drops, demonstrating the rapid attainment of peak concentrations despite the higher Cmax with THC/CBD-SE Powder.

**Fig. 3:**
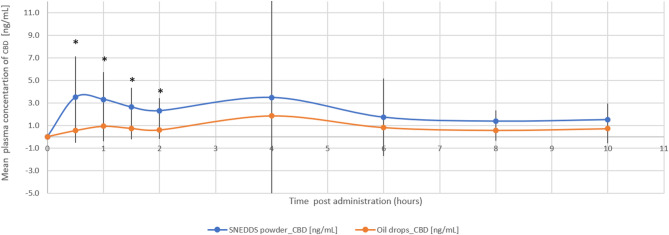
Mean plasma concentration of CBD over time following cannabis administration via THC/CBD-SE Powder (blue) and oil drops (orange) (*N* = 14). Vertical bars represent the standard deviation. *p < 0.05

**Fig. 4:**
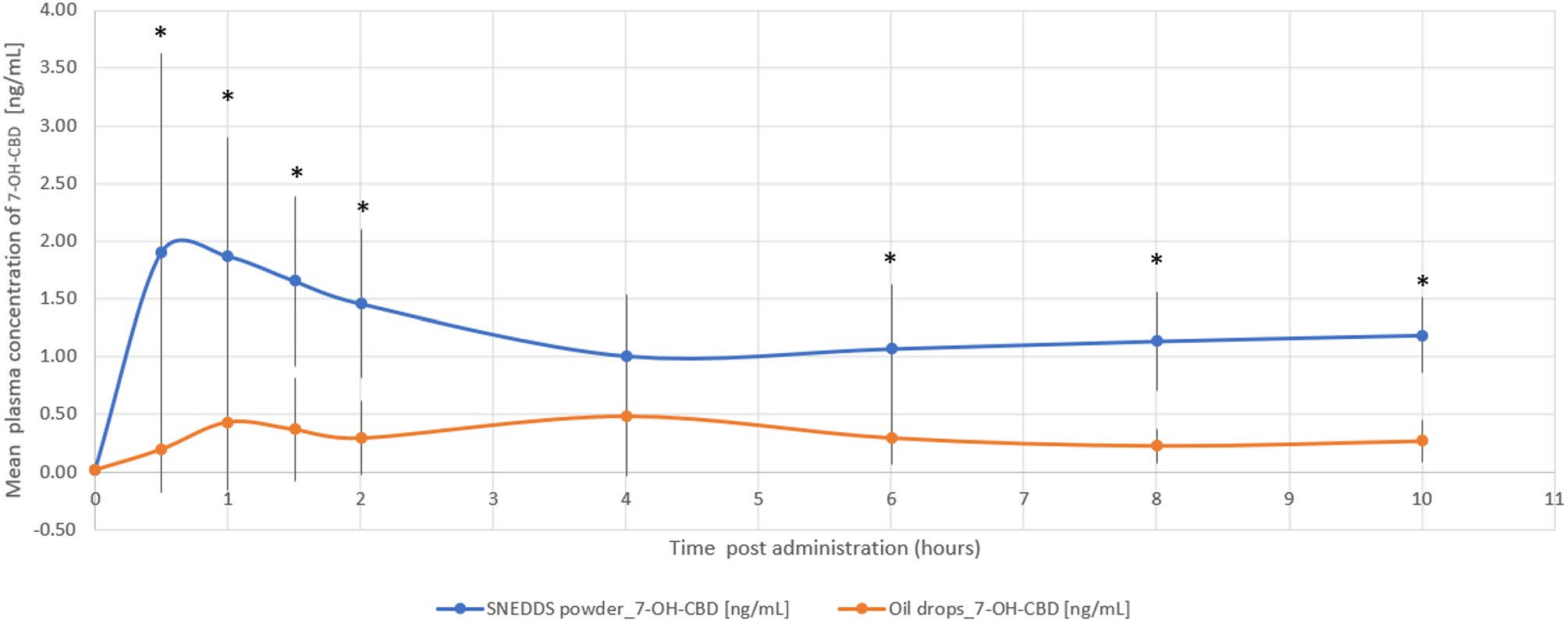
Mean plasma concentration of 7-OH-CBD over time following cannabis administration via THC/CBD-SE Powder (blue) and oil drops (orange) (*N* = 14). Vertical bars represent the standard deviation. *p < 0.05

The relative bioavailability of CBD and 7-OH-CBD was 2.3-fold (*p* = 0.018) and 3.2-fold (*p* = 0.004) higher with the THC/CBD-SE Powder, respectively. Table 2 provides a detailed summary of the pharmacokinetic parameters for CBD and 7-OH-CBD, underscoring the superior absorption and bioavailability of the THC/CBD-SE Powder compared to the oil drops. Figures 3 and 4 illustrate the mean plasma concentrations of CBD and 7-OH-CBD per hour post-administration for both formulations, further highlighting the enhanced pharmacokinetic profiles of the THC/CBD-SE Powder formulation.

**Table 2:** Pharmacokinetic parameters of CBD and 7-OH-CBD

Variable	THC/CBD-SE Powder Group (*N* = 14) Mean ± SD or %	Oil Drops Group (*N* = 14) Mean ± SD or %	*p*-value
CBD			
Cmax (ng/mL)	7.23 ± 8.54	3.3 ± 3.49	0.121
Tmax (h)	2.57 ± 3.17	4.57 ± 3.42	0.175
AUC (ng·h/mL)	20.23 ± 20.11	8.99 ± 8.77	**0.018**
Relative Bioavailability	100%	44%	-
7-OH-CBD			
Cmax (ng/mL)	2.38 ± 1.63	0.86 ± 0.56	**0.002**
Tmax (h)	1.11 ± 0.59	4.68 ± 3.38	**0.002**
AUC (ng·h/mL)	9.20 ± 5.62	2.92 ± 2.09	**0.004**
Relative Bioavailability	100%	32%	**-**

#### Cannabinoid clearance

Cannabinoid elimination was measured by clearance, defined as the volume of plasma from which THC and CBD are completely removed per unit of time. The study showed that the elimination of both THC and CBD from the body was slower with the SNEDDS method than with the oil drops, with clearance values of 0.32 ± 0.31 compared to 0.71 ± 0.49 for THC (*p* = 0.014) and 0.87 ± 0.75 compared to 3.93 ± 9.52 for CBD (*p* = 0.836), respectively.

### Clinical outcome

**Fig. 5:**
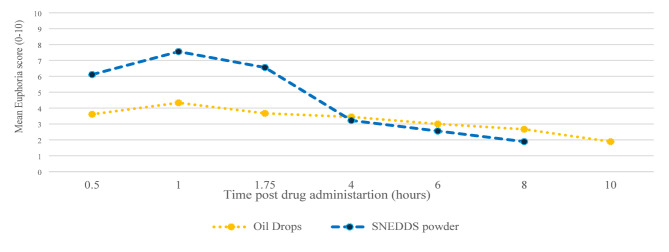
Euphoria scores post cannabis administration (*N* = 9)

Euphoria data were collected from 9 out of 14 (64%) study participants. Missing data points were extrapolated as the mean score between the preceding and subsequent scores. Both groups reported experiencing euphoria. The euphoria score was significantly higher up to 4 h post-cannabis administration via THC/CBD-SE Powder compared to oil drops (5.9 ± 2.8 vs. 3.8 ± 2.7, *p* = 0.0017). Afterward, the sensation was similar despite the higher availability of THC/CBD-SE Powder (*p* > 0.05). Figure 5 illustrates the euphoria scores post-cannabis administration.

### Safety

Both formulations were well-tolerated, with no serious adverse events reported. Mild to moderate adverse events included headache, dizziness, fatigue, decreased appetite, and increased pulse, all of which were transient and resolved without intervention. There were no significant differences in the incidence or severity of adverse events between the two formulations.

## Discussion and conclusions

The oral bioavailability of cannabinoids presents a well-documented challenge due to their lipophilic nature, limited gastrointestinal absorption, and extensive first-pass metabolism (Agurell et al. 1981; Gaston and Friedman 2017). Overcoming these obstacles is crucial to achieving desired therapeutic responses via oral delivery. Various advanced formulation strategies have been explored in the past to enhance the oral bioavailability of poorly water-soluble drug candidates, with several self-emulsifying formulations already on the market (Salawi 2022). Among these, certain formulations have previously demonstrated the potential to enhance the oral bioavailability of cannabinoids (Atsmon et al. 2018; Knaub et al. 2019; Cherniakov et al. 2017a, 2017b).

This study aimed to evaluate the pharmacokinetics and relative bioavailability of cannabinoids and their metabolites—specifically THC, 11-OH-THC, CBD, and 7-OH-CBD—administered via THC/CBD-SE Powder compared to a standard oil-based formulation. This novel SNEDDS-based powder achieves complete emulsification, ensuring stability, solubility, and consistent bioavailability.

The results confirmed that THC/CBD-SE Powder significantly enhanced the relative bioavailability of all tested cannabinoids compared to the oil-based formulation. Higher Cmax values and shorter Tmax times were observed for THC, CBD, and their active metabolites, indicating faster and more efficient absorption. Specifically, the relative bioavailability increased by 2.9-fold for THC, 2.5-fold for 11-OH-THC, 2.3-fold for CBD, and 3.2-fold for 7-OH-CBD. These improvements highlight the formulation’s ability to rapidly deliver active compounds into the bloodstream, potentially resulting in quicker therapeutic effects.

These outcomes are consistent with prior research on emulsion-based cannabinoid delivery systems. For instance, Berl et al. (2022) reported enhanced Cmax and faster Tmax with liquid emulsions compared to medium-chain triglyceride formulations. Similarly, Cherniakov et al. (2017a, 2017b) found that a capsule-based THC-CBD-piperine-pro-nanolipospheres delivery system resulted in a four-fold increase in Cmax and a 2.2-fold increase in AUC compared to Sativex^®^, an oromucosal spray. Additionally, Atsmon et al. (2018) demonstrated that an oral THC and CBD formulation via a self-emulsifying drug delivery system provided higher plasma Cmax and relative bioavailability compared to Sativex^®^. The consistency of these results supports the effectiveness of SNEDDS for cannabinoid delivery, underscoring its potential as a robust alternative to conventional cannabinoid delivery methods.

Interestingly, multiple peaks were observed in the concentration-time profiles, particularly for CBD at 4 h and THC at 8 h. These delayed secondary increases in systemic concentrations may be partly attributed to the lipophilic nature of cannabinoids, which enables their uptake into tissues such as adipose and subsequent redistribution into the plasma over time. This mechanism is consistent with prior observations and could also explain the smaller secondary fluctuations seen in the oil formulation (Huestis 2007). However, in the SNEDDS powder group, these multiphasic profiles were more pronounced. First, the rapid onset of absorption with the SNEDDS powder likely engages multiple absorption sites along the gastrointestinal tract, contributing to a more complex absorption profile. Second, the increased surface area of the self-emulsified nano-droplets may facilitate prolonged or sequential absorption across different intestinal segments, further accentuating the appearance of secondary peaks. Together, these mechanisms may help explain the appearance of multiphasic concentration-time profiles following oral cannabinoid administration, although further investigation would be needed to confirm their relative contributions.

The clinical implications of our findings are substantial. The enhanced relative bioavailability and faster onset of action provided by THC/CBD-SE Powder may support more efficient and timely therapeutic effects. This is particularly valuable in clinical settings where rapid relief is required, such as pain management or seizure control. While inhalation methods are known for their rapid absorption and almost immediate effects, they can also cause adverse effects, including respiratory issues and potential long-term lung damage (Tan and Sin 2018; Kaplan 2021; Tetrault et al. 2007; MacCallum and Russo 2018; Gracie and Hancox 2021; Tashkin 2013). THC/CBD-SE Powder, in contrast, provides a non-invasive oral alternative that delivers rapid systemic absorption without the risks associated with pulmonary route of administration. Furthermore, the enhanced relative bioavailability observed with THC/CBD-SE Powder suggests that therapeutic plasma concentrations may be achieved with smaller doses compared to conventional oil formulations. While this could potentially reduce the total amount of active compound administered and lower the risk of dose-dependent adverse effects, further studies are needed to confirm whether this translates to improved safety outcomes, particularly in vulnerable populations.

Despite these promising results, the study has limitations. The small sample size of 14 healthy participants limits the generalizability of the findings, and substantial inter-individual variability in pharmacokinetic parameters remains a challenge. This variability, frequently reported in cannabinoid studies (Atsmon et al. 2018; Knaub et al. 2019; Karschner et al. 2011; Huestis 2007), was partially mitigated by the crossover design, which controls for inter-individual differences. However, intra-individual variability could not be assessed due to the single administration of each formulation. These factors should be considered when applying these results in clinical practice, and further studies with larger, more diverse populations are needed to validate these findings.

In conclusion, THC/CBD-SE Powder significantly enhances the relative bioavailability and pharmacokinetic profile of cannabinoids compared to a standard oil-based formulation. The study results demonstrate that this technology provides a more efficient delivery method, leading to faster and more effective therapeutic outcomes. Its ability to deliver rapid therapeutic effects while reducing the risk of adverse outcomes positions it as a promising option for both acute and chronic conditions. Further research is warranted to validate its long-term safety and efficacy and to explore its potential in diverse patient populations. These findings provide a strong foundation for the development of THC/CBD-SE Powder as an effective and reliable cannabinoid delivery system.

## Data Availability

The datasets generated and analyzed during the current study are available from the corresponding author upon reasonable request.
